# The effect of different acute muscle contraction regimens on the expression of muscle proteolytic signaling proteins and genes

**DOI:** 10.14814/phy2.13364

**Published:** 2017-08-04

**Authors:** Satoru Ato, Yuhei Makanae, Kohei Kido, Kohei Sase, Naomi Yoshii, Satoshi Fujita

**Affiliations:** ^1^ Faculty of Sport and Health Science Ritsumeikan University Kusatsu Shiga Japan

**Keywords:** Contraction mode, proteolytic signaling, resistance exercise

## Abstract

Previous studies have reported that different modes of muscle contraction (i.e., eccentric or concentric contraction) with similar contraction times can affect muscle proteolytic responses. However, the effect of different contraction modes on muscle proteolytic response under the same force−time integral (FTI: contraction force × time) has not been investigated. The purpose of this study was to investigate the effect of different contraction modes, with the same FTI, on acute proteolytic signaling responses. Eleven‐week‐old male Sprague–Dawley rats were randomly assigned to eccentric (EC), concentric (CC), or isometric contraction (IC) groups. Different modes of muscle contraction were performed on the right gastrocnemius muscle using electrical stimulation, with the left muscle acting as a control. In order to apply an equivalent FTI, the number of stimulation sets was modified between the groups. Muscle samples were taken immediately and three hours after exercise. Phosphorylation of FoxO3a at Ser253 was significantly increased immediately after exercise compared to controls irrespective of contraction mode. The mRNA levels of the ubiquitin ligases, MuRF1, and MAFbx mRNA were unchanged by contraction mode or time. Phosphorylation of ULK1 at Ser317 (positive regulatory site) and Ser757 (negative regulatory site) was significantly increased compared to controls, immediately or 3 h after exercise, in all contraction modes. The autophagy markers (LC3B‐II/I ratio and p62 expression) were unchanged, regardless of contraction mode. These data suggest that differences in contraction mode during resistance exercise with a constant FTI, are not factors in regulating proteolytic signaling in the early phase of skeletal muscle contraction.

## Introduction

Over the past two decades, numerous researchers have reported that resistance training, with an emphasis on an eccentric contraction phase, results in both higher muscle mass and force‐gain compared with other contraction modes (i.e., concentric or isometric contraction) (Roig et al. 2009). In fact, eccentric contraction has been shown to induce greater levels of anabolic signaling (i.e., mTORC1 signaling) compared with other modes of contraction under identical muscle contraction times (Rahbek et al. 2014). Eccentric contraction can exert a greater force–time integral (FTI) than other contraction modes per unit time since it is able to generate a higher force. We, and another research group, have recently shown that different modes of resistance exercise (i.e., eccentric, concentric, and isometric contraction) with the same FTI, induced similar anabolic responses in a rodent experimental model (Garma et al. 2007; Ato et al. 2016). These studies indicated that the differences in anabolic response occurring following different modes of contraction are not due to the contraction mode per se, but rather to the magnitude of the FTI. Skeletal muscle mass is determined by muscle protein breakdown as well as by muscle protein synthesis (Bodine et al. 2001a,b; Masiero et al. 2009; Sandri 2013). However, only a few studies have focused on the regulation of muscle protein breakdown in response to acute resistance exercise (Glynn et al. 2010; Fry et al. 2013). Therefore, it is important to assess the effect of different contraction modes on muscle protein breakdown, and the activation of associated signaling pathways, in order to clarify whether the different contraction modes themselves directly affect protein metabolism following acute resistance exercise.

It is known that in skeletal muscle, protein degradation is regulated by four distinct cascades, namely the caspase cascade, the calpain pathway, the autophagy–lysosome system, and the ubiquitin–proteasome system. Caspase, a member of the cysteine protease family, is involved in apoptosis (i.e., programmable cell death), which results in nuclear disassembly (Janicke et al. 1998). For this reason, it is generally thought that the caspase pathway is not directly involved in myofibril protein breakdown. The calcium ion (Ca^2+^) sensitive calpain pathway is, however, thought to be very important in cytoskeletal structural remodeling in skeletal muscles (Huang and Forsberg 1998). Although little is known about the effect of resistance exercise on the calpain pathway, a previous study has reported that both calpain and calpastatin gene expression increased after acute resistance exercise in humans (Yang et al. 2006; Deldicque et al. 2008). The autophagy–lysosomal system is upregulated in energy deprivation states, such as during starvation and glycogen depletion, that result in an increase in the AMP/ATP ratio (Russell et al. 2014). In this low energy state, 5′ AMP‐activated protein kinase (AMPK) upregulates UNC‐51‐like kinase (ULK1) and subsequently increases isolation membrane and autophagosome formation (Li et al. 2013). Finally, the autophagosome binds to the lysosome, and induces protein degradation (Mizushima et al. 2011). In contrast, the mammalian target of rapamycin complex1 (mTORC1), which is composed mTOR, G*β*L, raptor, PRAS40, and DEPTOR as a large kinase complex, negatively regulates autophagy by ULK1 phosphorylation at Ser757 (Kim et al. 2011). Although resistance exercise induces both AMPK and mTORC1 activation in humans as well as animals, a previous study has reported that acute resistance exercise decreases the levels of a marker of isolation membrane formation (microtubule‐associated protein 1 light chain 3 beta; LC3B) in humans (Fry et al. 2013). Therefore, resistance exercise itself may downregulate autophagy. The ubiquitin–proteasome system is commonly associated with myofibril protein degradation, such as myosin, in skeletal muscle (Clarke et al. 2007; Lokireddy et al. 2012). Two specific E3 ubiquitin ligases, namely muscle specific RING finger 1 (MuRF1) and muscle atrophy F box (MAFbx, also known as atrogin‐1), have been identified in skeletal muscles (Bodine and Baehr 2014). These ligases polyubiquitinate target proteins and subsequently induce protein degradation via the 26S proteasome. These ligases have been shown to be regulated by the transcriptional factor forkhead box O3a (FoxO3a) in skeletal muscles (Sandri et al. 2004). Resistance exercise has been shown to negatively regulate FoxO3a via phosphorylation of its Ser253 residue (Fry et al. 2013). However, previous studies have demonstrated that an acute bout of resistance exercise increased MuRF1 mRNA and protein expression, and either decreased, or did not affect, MAFbx expression in humans (Mascher et al. 2008; Fry et al. 2013). Thus, there is currently no consensus on the effect of acute resistance exercise on ubiquitin ligase expression.

Only a limited number of studies have assessed the relationship between muscle protein degradation pathways and resistance exercise using different contraction modes (Nedergaard et al. 2007; Vissing et al. 2008; Stefanetti et al. 2014b). Although information about the effect of different contraction modes on the calpain pathway is limited, one study has demonstrated that eccentric contraction increased calpain gene expression to higher levels than concentric contraction during a bench stepping exercise in humans (Vissing et al. 2008). Ubiquitin ligase gene expression patterns have also been reported to be drastically different following different modes of contraction. For example, MAFbx gene expression was decreased after eccentric contraction, but was unchanged after concentric contraction (Nedergaard et al. 2007). It should be noted, however, that these studies used similar contraction times to assess differences caused by the different modes of contraction. Under identical muscle contraction times, the different modes of contraction produce different contraction‐induced FTI. In particular, eccentric contraction produces a higher FTI than concentric and isometric contraction (Eliasson et al. 2006; Rahbek et al. 2014; Ato et al. 2016). It is well recognized that the magnitude of the FTI influences negative regulators of proteolysis (i.e., Akt, mTORC1) (Russ 2008; Burd et al. 2010; Ato et al. 2016). Activated Akt phosphorylates FoxO3a at Ser253 and downregulates transcriptional activity (Sandri et al. 2004). Similarly, mTORC1 downregulates autophagy via ULK1 at Ser757 phosphorylation (Kim et al. 2011). We and others have previously observed that eccentric contraction induces higher Akt and mTORC1 activation compared with other contraction modes, under conditions when the contraction time was identical (Eliasson et al. 2006; Rahbek et al. 2014; Ato et al. 2016). In contrast, differences in contraction mode did not affect Akt and mTORC1 activity when the FTI was identical (Ato et al. 2016). Therefore, different FTI might modulate activation of the proteolytic system, including the autophagy and ubiquitin proteasome systems, following muscle contraction in response to different exercise modes.

The purpose of this study was to investigate the effect of different modes of muscle contraction on the proteolytic response, standardizing the contraction‐induced FTI.

## Methods

### Animals experimental procedures

The study protocol was approved by the Ethics Committee for Animal Experiments at Ritsumeikan University, Japan. Thirty male Sprague–Dawley rats, aged 10 weeks, were purchased from CREA Japan (Tokyo, Japan). The animals were acclimatized for 1 week in an environment maintained at 22–24°C with a 12–12 h light–dark cycle and received food and water ad libitum. The rats were randomly assigned to three experimental groups (*n* = 5/group/time point) designated as: eccentric contraction (EC), concentric contraction (CC), and isometric contraction (IC) groups. Subsequently, the rats were exercised after a 12 h overnight fast. The rats were sacrificed either immediately, or 3 h after, an acute bout of exercise, and the gastrocnemius muscle was removed immediately (within 5 min).

### Resistance exercise protocols

An acute resistance exercise protocol was carried out as previously described (Ato et al. 2016). Briefly, under inhaled isoflurane anesthesia (2%, Shinano Seisakusho, Tokyo, Japan), the lower right‐leg of each rat was shaved and cleaned with alcohol wipes. The rats were then positioned with their right foot on a footplate in the prone posture. The triceps surae muscle was stimulated percutaneously with disposable electrodes (Vitrode V, Ag/AgCl; Nihon Kohden, Tokyo, Japan), which were cut to measure 10 mm × 5 mm and connected to an electrical stimulator (SEN‐8203; Nihon Koden) and an isolator (SS‐104J; Nihon Koden). The right gastrocnemius muscle was exercised (3‐sec stimulation × 10 contractions, with a 7‐sec interval between contractions, per set, with 3‐min rest intervals). The number of sets was modified among the different contraction modes (EC, three sets; CC, four sets; IC, five sets) in order to standardize the groups to an equivalent force–time integral of five sets of isometric contractions. Output torque was measured (torque meter: Unipulse Corporation, Tokyo, Japan; A/D converter: CONTEC, Tokyo, Japan) continuously with a sampling rate of 500 Hz and analyzed using Microsoft Excel 2011.

### Western blot analysis

Western blot analysis was performed as reported previously (Ato et al. 2016). Briefly, powdered frozen muscle samples were homogenized in a homogenization buffer containing 100 mmol/L Tris–HCl (pH 7.8), 1% NP‐40, 0.1% SDS, 0.1% sodium deoxycholate, 1 mmol/L EDTA, and 150 mmol/L NaCl, with a protease and phosphatase inhibitor cocktail (Thermo Fisher Scientific, Waltham, MA) using a pestle homogenizer. Homogenates were centrifuged at 10,000*g* for 10 min at 4°C and the supernatant was removed, and the protein concentration of each sample was determined using the Protein Assay Rapid kit (Wako, Osaka, Japan). The samples were diluted in a Laemmli sample buffer (1.0% vol/vol *β*‐mercaptoethanol, 4.0% wt/vol SDS, 0.16 mol/L Tris HCl, pH 6.8, 43% vol/vol glycerol, and 0.2% wt/vol bromophenol blue) and boiled at 95°C for 5 min. Using 5–20% SDS‐polyacrylamide gels, both 20 and 50 *μ*g of protein were separated by electrophoresis and subsequently transferred to polyvinylidene difluoride membranes. After transfer, the membranes were washed in Tris‐buffered saline containing 0.1% Tween 20 (TBST), and membranes were then blocked with RAPIDBLOCK^TM^ SOLUTION (Amresco, Solon, OH) for 5 min at room temperature. After blocking, the membranes were washed and incubated overnight at 4°C with primary antibodies, including p‐AMPK*α* (Thr172, cat#2531), total AMPK (cat#2532), p‐ULK1 (Ser317, cat#12753), p‐ULK1 (Thr757, cat#14202), total ULK1 (cat#8054), LC3B (cat#2775), p‐FoxO3a (Ser253, cat#9466), total FoxO3a (cat#2497), calpastatin (cat#4146) (Cell Signaling Technology, Danvers, MA), calpain (S2 subunit) (cat#ab28241; Abcam, Cambridge, UK), p62 (cat#PM045) (Medical & Biological Laboratories, Aichi, Japan). The membranes were then washed again in TBST and incubated for 1 h at room temperature with the appropriate secondary antibodies. Chemiluminescent reagents (Luminata Forte Western HRP substrate; Merck Millipore, Darmstadt, Germany) were used to facilitate the detection of protein bands. Images were scanned using a chemiluminescence detector (ImageQuant LAS 4000; GE Healthcare, Buckinghamshire, UK). Band intensities were quantified using ImageJ 1.50f (National Institute of Health, Bethesda, MD).

### RT‐qPCR

Quantitative reverse transcription polymerase chain reaction (RT‐qPCR) was performed as previously described (Yokokawa et al. 2015). Total RNA was extracted from powdered frozen muscle samples using ISOGEN (Nippon Gene, Tokyo, Japan), in accordance with the manufacturer's instructions. Total RNA concentration was measured using a spectrophotometer (Nano Drop^TM^ 2000; Thermo Fisher Scientific, Waltham, MA) and 500 ng of RNA was reverse transcribed into cDNA using a ReverTra Ace qPCR RT Master Mix with gDNA Remover (Toyobo, Osaka, Japan).

Real‐time PCR was carried out using a RT‐qPCR master mix regent (KAPA SYBR^®^ Fast qPCR Kit; KAPA Biosystems, Wilmington, MA) and a real‐time PCR instrument (Applied Biosystems^®^ 7500 fast systems; Thermo Fisher Scientific, Waltham, MA) according to the manufacturer's instruction. The primers for RT‐PCR were purchased from Thermo Fisher Scientific. The following primer sequences were used; MuRF1 forward 5′‐TCGACATCTACAAGCAGGAA‐3′, MuRF1; reverse 5′‐CTGTCCTTGGAAGATGCTTT‐3′, MAFbx; forward 5′‐AGAAAAGCGGCACCTTCGT‐3′, MAFbx; reverse 5′‐CTTGGCTGCAACATCGCGTAGTT‐3′, GAPDH; (glyceraldehyde‐3‐phosphate dehydrogenase) forward 5′‐ CTCTCTGCTCCTCCCTGTTC‐3′, GAPDH reverse 5′‐ CGATACGGCCAAATCCGTTC‐3′. The housekeeping gene GAPDH was used as an internal control, and for normalization of cDNA concentrations.

### Measurement of plasma 3‐methyl histidine concentrations

Blood was drawn from the heart concomitant with muscle sampling (immediately and/or 3 h after exercise), collected into heparin tubes, and centrifuged at 1660*g* at 4°C; the plasma was stored at −80°C until analysis. In order to deproteinize the plasma, it was gradually thawed at 4°C, and 100 *μ*L of 15% sulfosalicylic acid was added to 100 *μ*L of plasma. Subsequently, the samples were vortexed and incubated for 20 min on ice. The samples were then centrifuged at 7000*g* at 5°C, for 10 min. The supernatant (170 *μ*L) was then added to an ultrafiltration tube (Merck Millipore, Merck Millipore, Darmstadt, Germany), and centrifuged at 15,000*g* at 5°C for 60 min. The filtered samples were applied to a High‐speed Amino Acid Analyzer (L8900; Hitachi High‐Technologies Corporation, Tokyo, Japan), to measure plasma 3‐methyl histidine (3‐MH) concentrations.

### Statistical analyses

Changes in the phosphorylation levels of signaling molecules, the 3‐MH concentration, and mechanical parameters (force–time integral/g body weight; BW) were analyzed by one‐way analysis of variance (ANOVA) and *t*‐test were performed, with a Benjamini and Hochberg false discovery rate correction for multiple comparisons when appropriate (JMP 10.0; SAS Cary, NC). All values are expressed as mean ± SE. The level of significance was set at *P* < 0.05.

## Results

The mean force–time integrals (FTI) obtained in this study were as follows: for EC; 13.59 ± 0.75 Nm・s/g BW, CC; 12.76 ± 0.68 Nm・s/g BW, IC; 11.89 ± 0.66 Nm・s/g BW. There was no statistical difference in mean FTI between the three different contraction modes used.

To examine the different muscle proteolytic pathways, we examined the expression of key proteolytic pathway proteins and mRNAs in muscle samples from rats exercised under the three different exercise modes, with muscle samples being taken either immediately after exercise, or after a 3 h recovery period.

**Figure 1:**
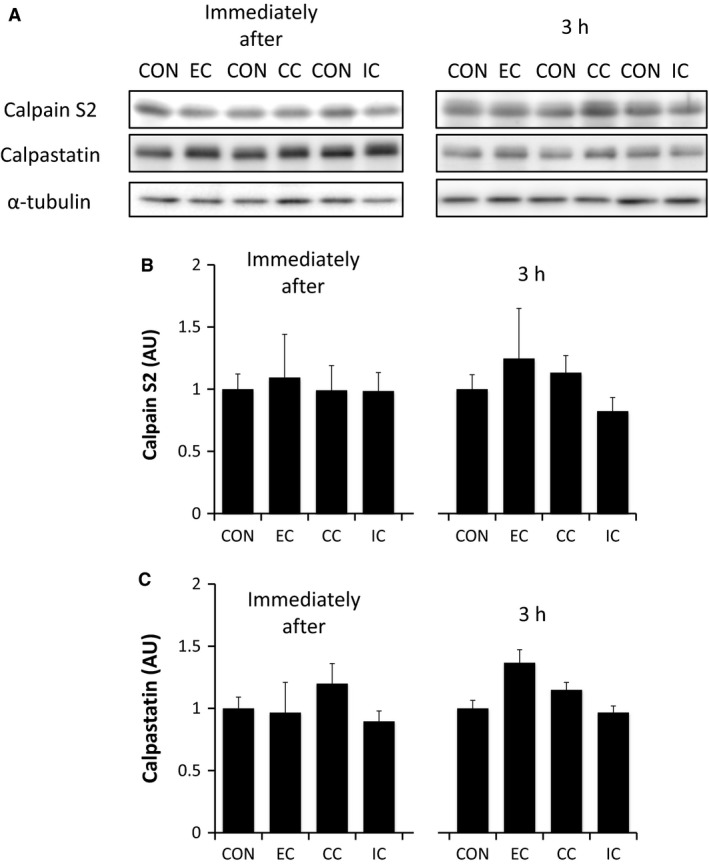
The effect of different resistance exercise regimens on the expression of muscle proteins that regulate the calpain pathway. Western blotting of calpain and calpastatin (A). Muscle samples were obtained immediately or 3 h after different resistance exercise regimens and assessed for calpain expression (B) (normalized to *α*‐tubulin) and calpastatin expression (normalized to *α*‐tubulin) (C). EC, eccentric contraction; CC, concentric contraction; IC, isometric contraction; CON, control muscle. Values are mean ± SE. AU, arbitrary unit.

With respect to the calpain pathway, we found that calpain S2 expression was unchanged immediately, as well as 3 h, after exercise in all contraction modes (EC, CC, IC) (Fig. 1B). Similarly, calpastatin expression was unaffected either immediately, or 3 h, after exercise in all contraction modes (Fig. 1C).

**Figure 2:**
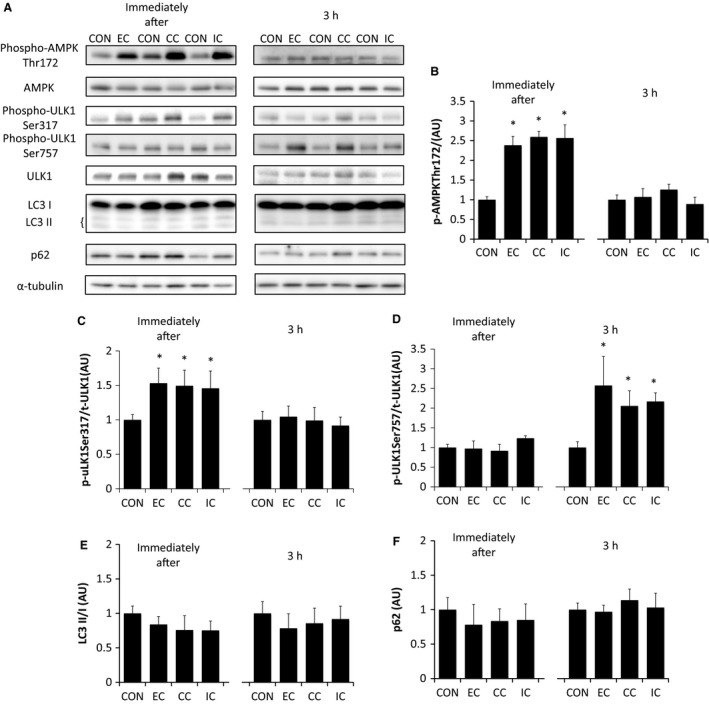
The effect of different resistance exercise regimens on the expression of muscle proteins that regulate autophagy. Western blotting of 5′ AMP‐activated protein kinase (AMPK), UNC‐51‐like kinase (ULK1), LC3B, and p62 (A). Muscle samples were obtained immediately or 3 h after different resistance exercise regimens and assessed for the levels of phosphorylated Thr172 AMPK (B), Ser317 phosphorylated ULK1 (C), and Ser757 phosphorylated ULK1 (D), all relative to total protein content. The ratio of LC3‐II to LC3‐I expression (E) and p62 expression (F) (normalized to *α*‐tubulin) were also determined. EC, eccentric contraction; CC, concentric contraction; IC, isometric contraction; CON, control muscle. Values are mean ± SE. *P < 0.05 versus CON. AU, arbitrary unit.

To examine the autophagy pathway, we examined the phosphorylation of AMPK, ULK1, as well as the LC3B‐II/I ratio and the levels of p62. AMPK Thr172 phosphorylation was significantly higher immediately after exercise compared to the control, regardless of the contraction mode (*P *<* *0.05 vs. CON). The phosphorylation of AMPK at Thr172 returned to control levels 3 h after exercise in all the contraction modes (Fig. 2B). Phosphorylation of ULK1 at Ser317 (the AMPK phosphorylation site) was significantly higher immediately after exercise compared with controls, regardless of the contraction mode (*P *<* *0.05 vs. CON). Similar to AMPK Thr172 phosphorylation, 3 h after exercise, the ULK1 Ser317 phosphorylation levels had returned to control levels across all the three different contraction modes (Fig. 2C). Phosphorylation of ULK1 at Ser757, a negative regulatory site phosphorylated by mTORC1, was unchanged immediately after exercise in all contraction modes. In contrast, the levels of ULK1 Ser757 phosphorylation increased significantly, compared to controls, 3 h after exercise in all three of the different contraction modes (*P *<* *0.05 vs. CON) (Fig. 2D) with similar degrees of ULK1 Ser757 phosphorylation found in the different contraction modes. The LC3B‐II/I ratio was unchanged, both immediately and 3 h after exercise, compared to controls, regardless of the contraction mode used (Fig. 2E). Similarly, the expression of p62 was also found to be unchanged, both immediately and 3 h after exercise (Fig. 2F).

**Figure 3:**
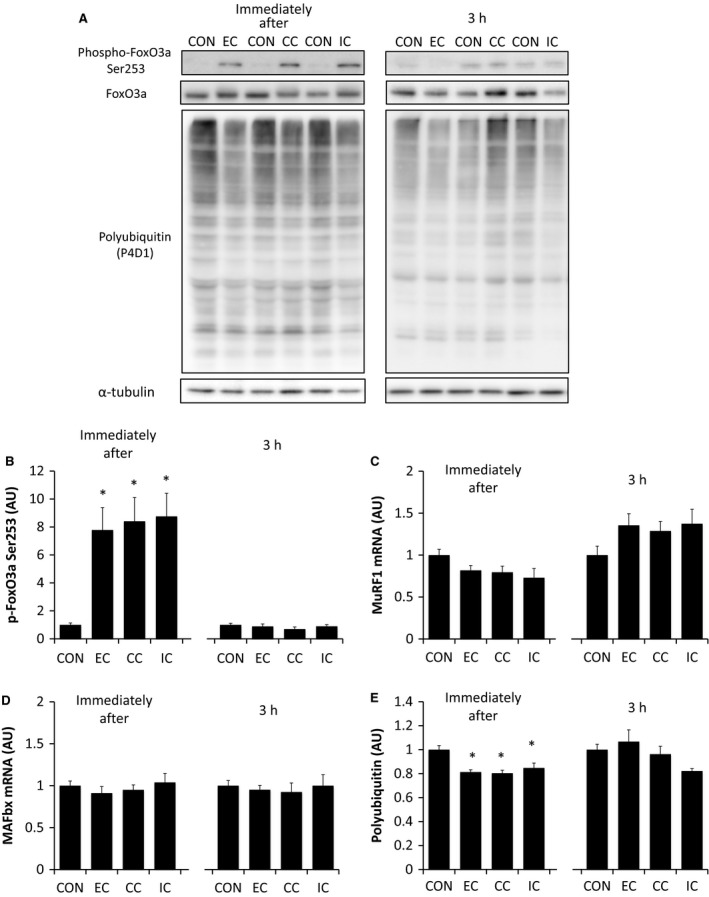
The effect of different resistance exercise regimens on the expression of muscle proteins that regulate the ubiquitin–proteasome system. Western blotting of FoxO3a, and polyubiquitin (A). Muscle samples were obtained immediately or 3 h after different resistance exercise regimens and assessed for the levels of Ser253 phosphorylated FoxO3a relative to total protein content (B), muscle specific RING finger 1 mRNA (C), muscle atrophy F box mRNA (D), and polyubiquitin expression (normalized to *α*‐tubulin) (E). EC, eccentric contraction; CC, concentric contraction; IC, isometric contraction; CON, control muscle. Values are mean ± SE. *P < 0.05 versus CON. AU, arbitrary unit.

To examine the ubiquitin–proteasome system we assessed phosphorylation of the transcription factor FoxO3a at Ser 253, as well as the mRNA levels of the E3 ubiquitin ligases MuRF1 and MAFbx. Phosphorylation of FoxO3a at Ser235 was significantly higher immediately after exercise in all of the contraction modes compared to controls (*P *<* *0.05 vs. CON), but there was no statistical difference in phosphorylation of FoxO3a at Ser235 between the different modes of contraction. At 3 h after exercise, the FoxO3a Ser235 phosphorylation level had returned to levels similar to controls in all the contraction modes (Fig. 3B). The mRNA levels of the E3 ubiquitin ligases, MuRF1 (Fig. 3C) and MAFbx (Fig. 3D) remained unchanged, both immediately and 3‐h after exercise, regardless of the contraction mode used. In contrast, the levels of polyubiquitin were lower immediately after exercise in all three of the different contraction modes compared to controls (*P *<* *0.05 vs. CON). There was no statistical difference in polyubiquitin levels between the different modes of contraction. Three hours after exercise, the polyubiquitin levels had returned to levels similar to the control in all three contraction modes (Fig. 3E).

**Figure 4:**
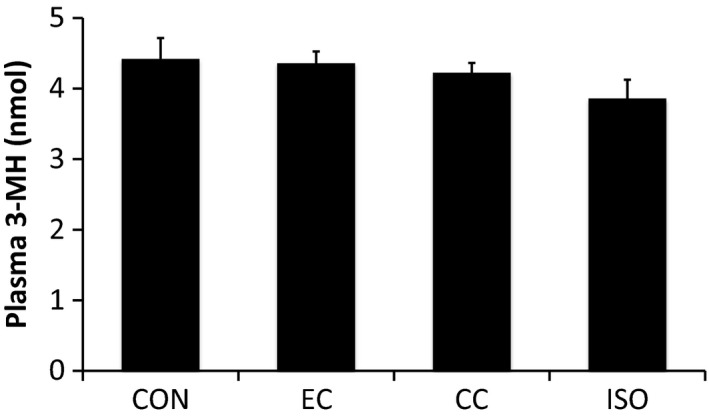
The effect of different resistance exercise regimens on plasma 3‐MH concentrations. EC, eccentric contraction; CC, concentric contraction; IC, isometric contraction; CON, control muscle. Values are mean ± SE.

In order to estimate alterations in whole body protein breakdown, we also assessed the plasma levels of 3‐methylhistidine (3‐MH). Plasma levels of 3‐MH were unchanged 3 h after exercise compared to controls in all three contraction modes (Fig. 4).

## Discussion

In this study, we investigated the effect of different contraction modes having similar FTI, on the intramuscular proteolytic response. The major findings of this study were that (1) resistance exercise‐like contraction increases the phosphorylation of both the inhibitory and the activatory phosphorylation sites on ULK1, whereas autophagy markers (LC3 II/I, p62) were unchanged after contractions regardless of the contraction mode used. (2) The transcription factor FoxO3a becomes phosphorylated on Ser253 immediately following exercise, whereas the mRNA levels of the ubiquitin ligases (MuRF1, MAFbx) were unchanged after exercise, regardless of the contraction mode used. (3) The levels of 3‐MH in the plasma were not affected after exercise, regardless of the contraction mode used.

5′ AMP‐activated protein kinase is known to be a critical regulator of autophagy (Kim et al. 2011). Muscle contraction during either resistance or endurance exercise increases AMPK phosphorylation immediately postexercise (Dreyer et al. 2006; Kido et al. 2016). In this study, we observed that AMPK phosphorylation increased immediately after exercise regardless of the contraction mode used. It is widely known that AMPK phosphorylation generally increases in proportion to muscle contraction time under conditions of constant contraction intensity (Rose et al. 2005; Miyamoto et al. 2007; Jensen et al. 2014). On the other hand, AMPK activity also increases with contraction force under a constant contraction time (Ihlemann et al. 1999). These data indicate that AMPK phosphorylation is increased in accordance with FTI (force or tension × time). Furthermore, since the phosphorylation level of acetyl‐CoA carboxylase, a target substrate of AMPK, is highly correlated with FTI (Rahnert and Burkholder 2013), this indicates that phosphorylation of AMPK increases in proportion to FTI. These data support our observation that the phosphorylation of AMPK at Thr172 was similar in all contraction modes having identical FTI.

ULK1 is a serine/threonine‐kinase, which regulates autophagy (Kim et al. 2011; Russell et al. 2013). Recent studies have reported that AMPK phosphorylates Ser317 in ULK1, and that the levels of ULK1 Ser317 phosphorylation were increased after aerobic exercise (Schwalm et al. 2015), although no data were presented examining the effect of acute resistance exercise. In this study, we found that ULK1 Ser317 phosphorylation increased immediately after resistance exercise‐like contractions, and returned to the basal state 3 h after exercise, independent of the contraction mode. In this regard, ULK1 Ser317 phosphorylation reflected the level of AMPK Thr172 phosphorylation, which would be expected since AMPK phosphorylates and regulates ULK1. Phosphorylation of a second residue in ULK1, namely Ser757, negatively regulates ULK1 activity via activation of mTORC1. A recent study has reported that resistance exercise increased ULK1 Ser757 phosphorylation in rodents (Ogasawara et al. 2016). In agreement with this previous study, we also observed an increase in phosphorylation of ULK1 at Ser757 3 h after exercise. Furthermore, the phosphorylation levels of ULK1 Ser757 did not differ between any of the contraction modes used in this study. We recently reported that muscle contraction induced by electrical stimulation increased mTORC1 activity (i.e., phosphorylation of p70S6K and/or 4E‐BP1) 3 h after exercise (Ato et al. 2016). Moreover, we have also reported that differences in contraction mode did not influence the magnitude of mTORC1 activation, as long as the FTI were matched. Taken together, the present data suggest that the increase in ULK1 Ser757 phosphorylation is reflective of mTORC1 activation. On the other hand, a recent animal study revealed that inhibition of mTORC1 using rapamycin does not affect the increased ULK1 phosphorylation seen after acute resistance exercise (Ogasawara et al. 2016). Although it has been proposed that the inhibitory effect of rapamycin on mTORC1 may not be complete (Thoreen and Sabatini 2009), this finding does raise the possibility that the increased ULK1 phosphorylation seen after resistance exercise may be due to an mTORC1‐independent mechanism, although this remains to be explored further.

Activated ULK1 regulates the autophagy process. LC3II is known to be an autophagy marker, which is formed when phosphatidylethanolamine binds to LC3I, and the resulting LC3II attaches to the isolation membrane (Kabeya et al. 2000). Therefore, the LC3II/I ratio is representative of the degree of isolation membrane formation. In this study, the LC3II/I ratio did not change after any mode of muscle contraction. Nevertheless, muscle contraction immediately increased the phosphorylation of ULK1 at Ser317 (a positive regulatory site), but did not immediately increase Ser757 phosphorylation (a negative regulatory site). Furthermore, we observed phosphorylation of ULK1 at Ser757, but not at Ser317, 3 h after exercise, whereas the LC3‐II/I ratio was unchanged 3 h after exercise. Recent animal experiments, using a similar experimental model, also showed that ULK1 Ser757 phosphorylation increased after resistance exercise, but did not affect the LC3‐II/I ratio (Ogasawara et al. 2016). In contrast, a decrease in the LC3‐II/I ratio was observed in humans, and this was maintained for up to 24 h after exercise (Fry et al. 2013). Consequently, the pathways that regulate isolation membrane formation in response to acute resistance exercise may differ between different species (human vs. rodent), or in the way the contraction was induced (i.e., voluntary vs. involuntary contraction). The expression of p62, a protein that directly bind to LC3, reflects the level of the autophagosome (Pankiv et al. 2007). In this study, the expression of p62 was not changed at any time point. However, a recent study reported another role of p62 in the recruitment of polyubiquitinated protein to the autophagosome via phosphorylation of the ubiquitin‐associated domain (Matsumoto et al. 2011). Therefore, further studies are needed in order to clarify the effect of resistance exercise and contraction mode on p62 activity and expression. Only a few previous studies have investigated the effect of resistance exercise on autophagy dynamics. The data generated in this study show that although resistance exercise increases ULK1 phosphorylation, there is no change in the LC3 and p62 proteins, suggesting that resistance exercise does not affect isolation membrane formation and autophagy. Additionally, our data show that differences in the mode of contraction, with the same FTI, do not affect autophagy in this rodent model.

The ubiquityn–proteasome system is another important regulator of proteolysis (Bodine et al. 2001a; Bodine and Baehr 2014). Previous studies have reported that an acute bout of resistance exercise increased the expression of ubiquitin ligase genes such as MuRF1 (Yang et al. 2006; Glynn et al. 2010; Fry et al. 2013). It is well recognized that the Akt‐FoxO3a pathway is involved in ubiquitin ligase expression (Sandri et al. 2004). We previously observed that the level of Akt phosphorylation did not differ between different modes of contraction having identical FTI (Ato et al. 2016). Therefore, we assumed that the changes in gene expression of ubiquitin ligases observed in response to different contraction modes in previous studies may have been caused using contraction modes having different FTI (i.e., identical contraction times but different contraction force). In this study, as expected, phosphorylation of FoxO3a was increased immediately after exercise regardless of the contraction mode. In contrast, a previous human study observed that phosphorylation of FoxO3a decreased 3‐24 h after resistance exercise, whereas Akt was activated at only 3 h postexercise (Fry et al. 2013). Although we cannot fully explain the reason for these conflicting results, the discrepancy may be due to species differences and/or the method used to induce muscle contraction. Additionally, while FoxO3a can translocate into the nucleus and regulate gene expression through its action as a transcription factor, it is retained in the cytosol and thereby inactivated as a result of phosphorylation at Ser253. Moreover, subcellular localization of FoxO3a is regulated by multiple phosphorylation sites other than Ser253 (Brunet et al. 1999). Therefore, subcellular localization is also an important factor, that in addition to protein abundance and/or protein modification (e.g., phosphorylation, methylation), should be considered when assessing FoxO3a activity. Further studies will be therefore be required to assess the changes in FoxO3a subcellular localization, along with protein expression changes, in response to the various contraction regimens used here.

Despite the fact that phosphorylation of FoxO3a increased, the mRNA levels of the transcriptional targets of FoxO3a, namely MuRF1 and MAFbx, were not changed immediately, or 3 h after of muscle contraction. On the other hand, study in humans has observed an increase in the gene expression of these ubiquitin ligases (Fry et al. 2013; Stefanetti et al. 2014b). This discrepancy might be attributed to the fact that Akt‐FoxO3a signaling was highly activated in this animal study compared to the human study. As stated previously, although FoxO3a was phosphorylated on Ser253 in this animal study, previous human studies have reported that phosphorylation of FoxO3a decreased after resistance exercise (Williamson et al. 2010; Fry et al. 2013; Stefanetti et al. 2014a), leading to an upregulation of FoxO3a transcriptional activity and a subsequent increase in ubiquitin ligase gene expression. Additionally, the differences in contraction mode have been reported to affect the mRNA expression of these ubiquitin ligases after resistance exercise in human. Eccentric contraction reduced MuRF1 and MAFbx mRNA expression compared with concentric contraction, under conditions of constant contraction times (Nedergaard et al. 2007; Stefanetti et al. 2014a). Nevertheless, in this study, similar levels of MuRF1 and MAFbx mRNA gene expression were observed when the FTI was identical. Since the expression of these ligase genes was unchanged in this study, we were unable to conclude whether different contraction modes affect the ubiquitin–proteasome system. There are several methodological limitations that need to be taken into consideration to interpret our study. First, a previous study has reported that following acute resistance exercise, ubiquitin ligase gene expression exhibits a biphasic change in expression that occurs over a 24‐h period following acute resistance exercise (Louis et al. 2007; West et al. 2012). In particular, MuRF1 mRNA increases early after exercise (~4 h), and then gradually decreases at later time points postexercise (~12 h). In this study, we analyzed only two very early time‐points after exercise (i.e., immediately and 3 h postexercise). Second, previous studies have reported that an alteration in ubiquitin ligase protein expression does not always reflect the degree of change in gene expression (Borgenvik et al. 2012; Stefanetti et al. 2014a,b). Therefore, further studies will be required to address changes in the protein levels of ubiquitin ligases. Third, in addition to abundance, the localization of proteolytic molecules in the cell (i.e., cytoplasmic, cytoskeletal, nuclear, or membrane) can be equally important. In particular, it is believed that ubiquitin ligases (e.g., MuRF) are located on titin in the thick filament of the sarcomere under basal conditions, and induce proteolysis once they are released (Lange et al. 2005; Ottenheijm et al. 2011; Kruger and Kotter 2016). Consequently, future studies should focus on addressing these limitations in order to more completely clarify the role of ubiquitin ligases under different contraction regimens. It is noteworthy that intracellular polyubiquitin levels were lower immediately after exercise. Since ubiquitin ligase gene expression was unchanged, this suggests that the ubiquitin system was not activated. Therefore, together these data suggest that the polyubiquitinated protein was digested in the proteasome. A recent in vitro study demonstrated that mTORC1 inhibition increased both the ubiquitin system and the proteasomal protein degradation (Zhao et al. 2015). In addition, mTORC1 activity has been shown to be acutely reduced immediately following resistance exercise in both humans and rodents (Dreyer et al. 2006; Ato et al. 2016). However, in this study we did not assess proteasome activity. Therefore, a further study is warranted to understand the mechanism of the acute decrease in polyubiquitinated proteins following resistance exercise and its potential physiological function.

The calpain pathway is involved in cytoskeletal remodeling. A previous study has reported an increase in calpain gene expression 72 h after acute resistance exercise in human (Deldicque et al. 2008). Furthermore, calpain and calpastatin gene expression increased 24 h after eccentric contraction, but not after concentric contraction, in a bench stepping exercise (Vissing et al. 2008). In disagreement with these previous studies, we did not observe any changes in the protein expression of calpain and calpastatin. In this study, we assessed calpain and calpastatin expression immediately and 3 h after muscle contraction, whereas the previous studies observed changes in calpain and calpastatin at a much later time point (~72 h) (Deldicque et al. 2008). Thus, the inconsistency between these results could be due to the different sampling times. In support of this, a typical resistance exercise regimen, including concentric and eccentric contraction (e.g., 65% 1RM knee extension), was shown not to affect calpain and calpastatin gene expression until 24 h after exercise in humans (Yang et al. 2006; Deldicque et al. 2008). Further detailed time course studies will be therefore be required to fully elucidate the effect of different contraction modes on the calpain–calpastatin pathway.

Finally, we investigated whether different contraction modes affected muscle protein breakdown. 3‐methylhistidine (3‐MH) is produced by the methylation of myofibrilar proteins (actin and myosin) (Johnson and Perry 1970), and its concentration in plasma is used as a biomarker of muscle protein breakdown (Sugden and Fuller 1991). In this study, plasma 3‐MH concentrations did not change at 3 h after exercise, regardless of contraction mode. This result suggests that, in the early phase following exercise, resistance exercise failed to increase muscle protein breakdown, whereas at the same time, the signaling pathways related to protein breakdown were altered. However, a previous human study found that resistance exercise increased the fractional protein breakdown rate, but did not affect plasma 3‐MH concentrations 3 to 24 h after exercise (Phillips et al. 1997). Thus, it is likely that muscle protein breakdown may not be adequately detected simply by measuring plasma 3‐MH levels, so an alternate method of measuring muscle protein breakdown is needed.

As previously discussed, when the contraction time is constant, the FTI level varies because the maximal torque output differs between the various contraction modes. Consequently, eccentric contraction should produce higher levels of mTORC1 activation under constant contraction time. In contrast, similar levels of anabolic signaling activation (i.e., mTORC1 signaling) has been observed using different contraction modes, when the contraction time was adjusted so that the FTI constant (Ato et al. 2016). In this study, under conditions of a constant FTI, although an acute bout of resistance exercise affected signaling molecules associated with autophagy and the ubiquitin–proteasome system (i.e., ULK1, FoxO3a), protein breakdown (i.e., lysosomal and proteasomal protein degradation) was not affected by resistance exercise regardless of the contraction mode. Our present data and a recent acute exercise study suggest that differences in the mode of contraction mode may not influence the magnitude of muscle hypertrophy when the FTI is matched. Several human and animal studies that assessed the magnitude of muscle hypertrophy using different contraction mode with identical FTI support our result concerning acute exercise (Moore et al. 2012; Eftestol et al. 2016). In contrast, several previous studies have reported that differences in magnitude of FTI due to divergence in contraction regimens do not necessarily reflect the magnitude of hypertrophy in rodent or human models (Adams et al. 2004; Franchi et al. 2015). Therefore, elements other than FTI that are included in the different contraction regimens may influence overall protein metabolism involved in chronic muscle hypertrophy, for example, nuclear accretion and/or remodeling of the extracellular matrix, amongst others.

In conclusion, our present data suggest that differences in contraction mode during resistance exercise do not have an effect on proteolytic signaling in the early phase after contraction in skeletal muscle.

## Conflict of Interest

The authors declare no conflicts of interest.
